# Exposure to perfluoroalkyl and polyfluoroalkyl substances and pediatric obesity: a systematic review and meta-analysis

**DOI:** 10.1038/s41366-023-01401-6

**Published:** 2023-10-31

**Authors:** Brianna Frangione, Sapriya Birk, Tarek Benzouak, Laura A. Rodriguez-Villamizar, Fatima Karim, Rose Dugandzic, Paul J. Villeneuve

**Affiliations:** 1 Department of Neuroscience, Carleton University, K1S 5B6 Ottawa, Canada; 2 Faculty of Medicine, McGill University, H3A 0G4 Montreal, Canada; 3 Faculty of Health, Universidad Industrial de Santander, 680002 Bucaramanga, Colombia; 4 Health Canada/Santé Canada, K1A 0K9 Ottawa, Canada; 5 CHAIM Research Centre, Carleton University, K1S 5B6 Ottawa, Canada

**Keywords:** Risk factors, Obesity, Paediatrics

## Abstract

**Introduction:**

Perfluoroalkyl and polyfluoroalkyl substances (PFAS) are potentially obesogenic for children. We undertook a systematic review to synthesize this literature and explore sources of heterogeneity in previously published epidemiological studies.

**Methods:**

Studies that collected individual-level PFAS and anthropometric data from children up to 12 years of age were identified by searching six databases. We excluded studies that only evaluated obesity measures at the time of birth. A full-text review and quality assessment of the studies was performed using the Office of Health Assessment and Translation (OHAT) criteria. Forest plots were created to summarize measures of association and assess heterogeneity across studies by chemical type and exposure timing. Funnel plots were used to assess small-study effects.

**Results:**

We identified 24 studies, of which 19 used a cohort design. There were 13 studies included in the meta-analysis examining various chemicals and outcomes. Overall prenatal exposures to four different types of PFAS were not statistically associated with changes in body mass index (BMI) or waist circumference. In contrast, for three chemicals, postnatal exposures were inversely related to changes in BMI (i.e., per log10 increase in PFOS: BMI z-score of −0.16 (95% CI: −0.22, −0.10)). There was no substantial heterogeneity in the reported measures of association within prenatal and postnatal subgroups. We observed modest small-study effects, but correction for these effects using the Trim and Fill method did not change our summary estimate(s).

**Conclusion:**

Our review found no evidence of a positive association between prenatal PFAS exposure and pediatric obesity, whereas an inverse association was found for postnatal exposure. These findings should be interpreted cautiously due to the small number of studies. Future research that can inform on the effects of exposure mixtures, the timing of the exposure, outcome measures, and the shape of the exposure-response curve is needed.

## Introduction

Worldwide, the prevalence of pediatric obesity has increased substantially between 1980 and 2015 [1]. The subsequent health impacts are diverse and substantial, and include physical and psychological health effects, including decreased self-esteem [2]. Furthermore, children with obesity are more likely to be obese in adulthood, increasing the risk of developing chronic diseases such as cardiovascular disease, cancer, and diabetes [2]. Additionally, there is evidence that pediatric obesity increases the risk of premature mortality [3].

Several modifiable risk factors have been identified as contributors to the increase in pediatric obesity. These include a lack of participation in recreational activities, high-fat diets, and increased screen time [2]. In addition to these factors, there has been an increased recognition that environmental exposures influence the rates of pediatric obesity [4, 5]. It follows that regulations that decrease exposure to environmental obesogens provide opportunities to reduce rates of obesity at a population level.

One class of such environmental exposures is perfluoroalkyl or polyfluoroalkyl substances (PFAS). These are synthetic chemicals that, because of their chemical properties, are commonly used in various products, including surfactants, cosmetics, food packaging, firefighting, waxes, non-stick cookware, and paints [6]. Perfluorooctanoic acid (PFOA) and perfluorooctane sulfonate (PFOS) are the most common environmental PFASs as they were the first manufactured compounds [7]. PFAS are ubiquitous and found in water, soil, and sediments, and due to their structural stability, they do not degrade in the environment, resulting in an ever-increasing accumulation [8]. Studies have also shown evidence of their accumulation in plants, wildlife, and humans [7]. Evidence suggests that in humans, PFAS accumulates in the blood, liver, brain, skeletal tissues, fetal lungs, and placenta [9, 10]. PFAS are broadly reported to have adverse effects on human health outcomes, including childhood growth and obesity [11].

There are several possible mechanisms whereby PFAS increase the risk of obesity [12]. They have been associated with genetic and hormonal factors linked to obesity [13]. PFAS act as endocrine-disrupting chemicals and several harmful health effects may be associated with the disturbance of hormone homeostasis [14]. Exposure to PFAS can trigger several molecular initiation pathways through interactions with nuclear transcription factors [15]. For example, a study conducted with mice by Bjork and colleagues reported interactions between PFOA and peroxisome proliferator-activated receptor alpha (PPARα), estrogen receptor α [16], and PPARγ [17]. Interactions with these major transcription factors have the potential to trigger multiple biochemical pathways and affect several biological systems simultaneously [18]. Adipose tissue metabolism is modulated by hormones, including sex hormones [19]. The effect of endocrine-disrupting chemicals is widespread and may affect lipogenesis, lipolysis, and adipogenesis [20]. A recent cohort study reported a statistically significant inverse association between PFAS concentrations and resting metabolic rate, which may contribute to increased weight gain or interference with weight loss [12]. Mechanistically, the timing of the exposure is likely to be important. It has been suggested that a fetus is more susceptible to PFAS exposure in-utero than postnatal exposure because PFAS can easily pass through the placenta and enter the fetus’s stream [21, 22]. Additionally, exposure to these endocrine-disrupting chemicals may result in epigenetic changes, which can result in altered adipocyte metabolism and function later in life [23].

While there are uncertainties about the biological mechanisms and the timing of the exposure whereby PFAS increases the risk of pediatric obesity, these exposures are modifiable. There is an increasing awareness and agreement that PFAS must be regulated on multiple levels to minimize environmental and human health impacts [24]. For example, the company 3M, a significant contributor to PFOS production, has agreed to phase out these chemicals within its products and replace them with more environmentally sustainable options [25]. Since then, several governmental bodies worldwide have acknowledged the potential impacts and adopted broad regulatory measures for their production and use [24]. Additionally, several remediation approaches have been implemented to help reduce the existing PFAS concentrations within contaminated soil and water supplies; however, this review will not focus on these emerging technologies [24, 26].

In a 2014 systematic review by Johnson and colleagues, they performed a meta-analysis of nine studies and found that increasing prenatal PFOA exposure was associated with an 18.9 g (95% CI: −29.8, −7.9) decrease in birth weight [27]. In 2022, Stratakis and colleagues [28] conducted a meta-analysis of studies assessing prenatal exposure to persistent organic pollutants and did not find statistically significant associations between PFAS and pediatric obesity. The strength of the association between PFAS and obesity varies across studies and type of chemical [28–32], and several studies have reported results of association since then [33–37]. Furthermore, previous systematic reviews have primarily focused on prenatal exposure despite studies reporting postnatal exposure to PFAS and pediatric obesity [34, 35, 38]. This systematic review and meta-analysis aims to synthesize recent evidence regarding prenatal and postnatal exposure to PFAS and pediatric obesity. The heterogeneity within the literature by chemical type and exposure timing will also be evaluated.

## Methods

### Protocol and registration

This systematic review was conducted in adherence to the Preferred Reporting Items for Systematic Reviews and Meta-Analyses (PRISMA) statement for the reporting [39] and Meta-analysis Of Observational Studies in Epidemiology (MOOSE) guidelines [40]. The study protocol and MEDLINE search strategy were submitted to the International Prospective Register of Systematic Reviews on February 23rd, 2022 and underwent full registration on March 28th, 2022 (Registration Number: CRD42022312862).

### Search strategy

We searched MEDLINE, Embase, PsycINFO, CINAHL, Web of Science, and Cochrane Central on February 26th, 2022. The search strategy was developed by one author (T.B.). No restrictions relating to study design or location were imposed within the search. Gray literature was not considered. Only human studies published from the year 2000 onwards were included. A manual search of citations from included papers was conducted to identify additional relevant literature.

Rayyan (QCRI) software [41] was used to manage screening procedures. One reviewer (B.F.) screened every title, abstract, and full text. A second independent reviewer (S.B. or F.K.) subsequently screened each title, abstract, and full text. Any discrepancies were resolved by a third screener (T.B.).

### Selection criteria

Our inclusion criteria allowed for case-control, cohort, and cross-sectional studies. All studies were required to have individual measures of exposure (i.e., PFAS) and outcome (i.e., obesity). Studies were required to meet the following criteria for eligibility within this systematic review: (i) PFAS exposure assessment during in-utero or early childhood periods of development; (ii) Weight assessments based on objective measurements; (iii) Weight-related measures performed during childhood developmental periods (i.e., 1–12 years of age). In addition, all studies had to report a measure of association that described the association between PFAS and a measure of obesity.

We also applied several exclusion criteria. For example, we did not include studies that did not quantitatively assess PFAS exposure. Furthermore, we did not consider studies that only assessed obesity/weight at birth, as birthweight was the focus of the systematic review by Johnson et al. [27]. In addition, we restricted to papers published in either English or French. Finally, we excluded studies of animals, simulated data, or commentaries.

### Data extraction

The data extracted from the identified studies were entered into a standardized Excel spreadsheet. This extraction sheet was developed a priori and reviewed by all authors. One author (B.F.) initiated and completed data extraction, which was then cross-validated by a second author (S.B.). We extracted key characteristics from each study, including study design, exposure source(s), type of outcome(s), and relevant measures of association.

### Quality of evidence

The risk of bias for studies meeting inclusion was assessed independently by two reviewers (B.F. and L.R.) using the Office of Health Assessment and Translation (OHAT) risk of bias tool [42]. Any discrepancies were discussed between the reviewers. Following OHAT methodology, studies were identified as belonging to Tier 1, reflecting a low risk of bias, Tier 2 in the presence of plausible risk of bias or Tier 3, representing a high risk of bias. To be categorized as a Tier 1 study, key elements were required to be rated as either “definitely low” or “probably low” risk of bias. In contrast, a study was categorized as belonging to Tier 3 when key domains were deemed sufficiently biased to affect the interpretation of study outcomes. This occurred if key domains were rated as “definitely high” or “probably high” risk of bias while concurrently having most other study characteristics deemed as “definitely high” or “probably high” risk of bias. Tier 2 was used to identify studies that met neither the criteria for Tier 1 nor Tier 3 quality assessment categories.

### Statistical analysis

The inverse variance method was used for the meta-analysis to assign study weight and create forest plots [43]. To account for within and between study variance in the measures of association, random-effects models were used to generate a summary measure of association across all studies. When studies reported effect estimates for two time points (i.e., measures at ages 4 and 7), they were combined using a fixed-effects meta-analysis [32, 44]. However, this was not done for studies that reported prenatal and postnatal exposure measurements, and they were instead included in the subgroup analyses. We acknowledge that considering results from the same study as if they originated from distinct studies may lead to underestimating the heterogeneity between studies. We wanted to evaluate whether associations differed by the timing of the exposure (prenatal vs. postnatal); therefore, separate models were fit for these two exposure periods. The *I*^*2*^ statistic [45] was used to assess the relative between-study heterogeneity, and we considered *I*^*2*^ > 40% moderate and *I*^*2*^ > 75% high heterogeneity. The τ^2^ statistic assessed the absolute between-study heterogeneity [46]. We assessed publication bias using funnel plots and formally tested for the presence of this bias using Egger’s test [47]. Where necessary, the Trim and Fill method [48] was used to correct the summary measure of association for publication bias. All analyses were conducted using STATA version 17 [49].

Pre-specified subgroup comparisons were further conducted to examine the influence of biological sex, age, and temporal characteristics of measurements (e.g., PFAS measurements in-utero (prenatal) or early childhood (postnatal) outcome).

Due to highly skewed exposure measurements, most studies had log10 transformed the PFAS concentrations. As such, we have reported the results similarly, as a log10 unit increase in PFAS concentration. If the study had used another transformation, such as log2, this was converted into log10, when applicable. Studies that reported increases in standard deviations (SD), or interquartile ranges (IQR), could not be converted and were excluded from the meta-analysis.

## Results

**Fig. 1:**
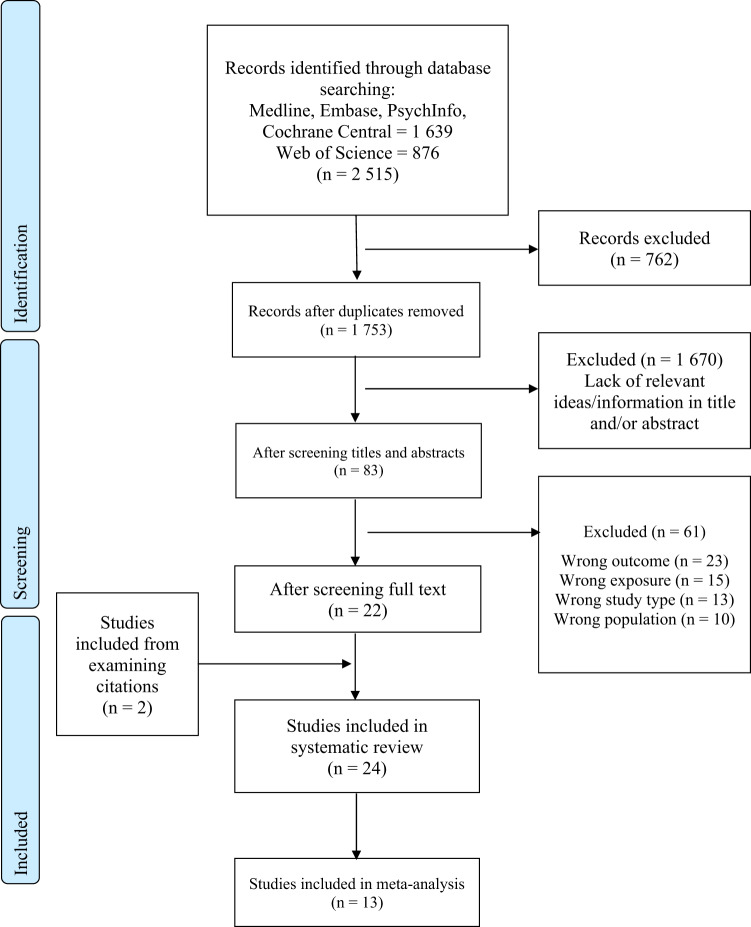
PRISMA flow diagram detailing search strategy.

We identified 24 studies that reported the association between exposure to PFAS and pediatric obesity (Fig. 1). Of these papers, 19 used a cohort study design, while the remaining studies were cross-sectional (*n* = 3) or case-control (*n* = 2) studies. The two most studied outcomes were changes in body mass index (BMI) z-scores (*n* = 17) and waist circumference (WC) z-scores (*n* = 6). In addition, changes in BMI (*n* = 3) and WC (*n* = 4) were examined; however, these studies were not included in our meta-analysis as they were not corrected for age and sex like their z-score counterparts.

Several studies examined exposure to multiple PFAS (see Table 1). For example, there were 24 studies which examined PFOA exposure, 23 studies on PFOS exposure, 17 studies on perfluorohexanesulfonic acid (PFHxS) exposure, 16 studies on perfluorononanoic acid (PFNA) exposure, and several studies on perfluorodecanoic acid (PFDA) (*n* = 3), perfluoroundecanoic acid (PFUnDA) (*n* = 4), perfluorododecanoic acid (PFDoA) (*n* = 1), perfluoroundecanoic acid (PFUA) (*n* = 1), and perfluorobutanesulfonic acid (PFBS) (*n* = 1) exposures.

**Table 1:** Descriptive characteristics of included studies.

Authors	Location	Study Design	Sample Size	Ages (years)	Timing of Exposure	Outcome	Exposure	Measure of Association	Adjusted Confounders
Andersen et al. [50]	Denmark	Prospective cohort	1400	7	Prenatal	BMI z-score WC z-score	PFOS PFOA	Slope^a^	Child’s age, maternal age, parity, pre-pregnancy BMI, smoking, socioeconomic status, gestational week at blood drawing, gestational weight gain, birth weight, duration of breastfeeding, weight at 5 m and 12 m
Bloom et al. [35]	USA	Prospective cohort	803	4–8	Prenatal	BMI z-score WC z-score	PFOS PFOA PFHxS PFNA PFUnDA	Slope	Maternal age at the time of collection, self-reported pre-pregnancy BMI, education, race/ethnicity, plasma cotinine concentration as an indicator of environmental tobacco smoke exposure
Braun et al. [29]	USA	Prospective cohort	204	2–8	Prenatal	BMI z-score WC z-score	PFOS PFOA PFHxS PFNA	Slope^a^ Relative risk	Maternal race, age, education, marital status, employment, household income, maternal depressive symptoms at 16 weeks’ gestation, BMI at 16 weeks’ gestation, parity, serum cotinine (a biomarker of tobacco smoke exposure), frequency of fresh fruit/vegetable and fish consumption during pregnancy, prenatal vitamin use
Canova et al. [36]	Italy	Cross-sectional	2693	8–11	Postnatal	BMI z-score	PFOS PFOA PFHxS	Slope^a^	Age, gender, country of birth, food consumption, physical activity, salt intake, smoking status (adolescents), time lag between the beginning of the study and the date of enrollment
Chen et al. [55]	China	Prospective cohort	404	5	Prenatal	BMI WC	PFOS PFOA PFHxS PFNA PFDA PFUA PFBS	Slope	Maternal age, ethnicity, education, pre-pregnancy BMI, smoking, paternal smoking during pregnancy, alcohol intake during pregnancy, parity, gestational age, infant sex
Fassler et al. [57]	USA	Cross-sectional	353	6–9	Postnatal	BMI z-score	PFOS PFOA PFHxS PFNA	Slope^a^	Race/ethnicity, age
Gross et al. [30]	USA	Case-control	52 cases 46 control	18 m	Prenatal	Weight for length z-score	PFOS PFOA PFHxS PFNA	Odds ratio	Maternal age, maternal education, prenatal depressive symptoms, pre-pregnancy BMI, gestational age, parity, intervention status
Hartman et al. [53]	UK	Prospective cohort	359	9	Prenatal	BMI WC	PFOS PFOA PFHxS PFNA	Slope	Maternal pre-pregnancy BMI, educational status, and PFNA models also adjusted for maternal smoking status
Horikoshi et al. [33]	Japan	Prospective cohort	597	5.5	Prenatal	BMI z-score	PFOS PFOA	Slope^a^	Infant sex, maternal age at delivery, pre-pregnancy BMI, maternal education, household income, maternal smoking during pregnancy, parity, gestational age, duration of breastfeeding
Hoyer et al. [31]	Greenland Ukraine	Prospective cohort	571 612	5–9	Prenatal	BMI z-score	PFOS PFOA	Slope^a^ Relative risk	Maternal age at birth, parity, maternal smoking during pregnancy, maternal education, pre-pregnancy BMI
Karlsen et al. [38]	Denmark	Prospective cohort	349	5	Postnatal	BMI z-score	PFOS PFOA PFHxS PFNA PFDA	Slope^a^	Maternal nationality, age at delivery, pre-pregnancy BMI, gestational weight gain, parity, smoking during pregnancy, maternal fish intake during pregnancy, type of delivery, child sex, birth weight
Lauritzen et al. [56]	Sweden Norway	Prospective cohort	412	5	Prenatal	BMI z-score	PFOS PFOA	Slope^a^	Maternal age, maternal pre-pregnancy BMI, maternal education, maternal smoking status at conception, breastfeeding duration, the inter-pregnancy interval between the last two children, maternal weight gain from conception to gestational week 17
Lee et al. [61]	Korea	Prospective cohort	361	2	Postnatal	Change in height and weight	PFOS PFOA PFHxS PFNA PFDA PFUnDA	Slope	Height at two years of age: age, gender, average parental height, height change from birth to 2 years of age was adjusted by the duration of breastfeeding Weight at two years of age: age, gender, parental BMI, and weight change from birth to 2 years of age was adjusted by the duration of breastfeeding
Manzano-Salgado et al. [32]	Spain	Prospective cohort	1086	4 & 7	Prenatal	BMI z-score WC z-score	PFOS PFOA PFHxS PFNA	Slope^a^	Maternal country of birth, parity, previous breastfeeding, maternal age, pre-pregnancy BMI, child sex and age
Marks et al. [37]	UK	Prospective cohort	301	19 m	Prenatal	Weight for age z-score	PFOS PFOA PFHxS PFNA	Slope	Gestational age at sample collection, age at measurement, maternal age at delivery, pre-pregnancy BMI, maternal education, parity, smoking during pregnancy
Martinsson et al. [51]	Sweden	Case-control	354 cases 694 control	4	Prenatal	BMI z-score	PFOS PFOA PFHxS PFNA	Odds ratio	Maternal smoking during pregnancy, birth weight, economic strain, being a tenant, parental obesity
Mora et al. [54]	USA	Prospective cohort	1882	3–11	Prenatal	BMI z-score WC	PFOS PFOA PFHxS PFNA	Slope	Maternal age, race/ethnicity, education, parity, pre-pregnancy BMI, timing of blood draw, household income, child sex and age at outcome assessment
Papadopoulou et al. [34]	Europe	Prospective cohort	1101	8	Prenatal Postnatal	WC z score	PFOS PFOA PFHxS PFNA PFUnDA	Slope^a^	Cohort, maternal age, parity, maternal education, pre-pregnancy BMI, child ethnicity, age at examination, sex
Pinney et al. [44]	USA	Prospective cohort	333	6–11	Postnatal	BMI z-score	PFOA	Slope^a^	Race/ethnicity, parent education, age at examination, average kilocalories in prior 12 months, hours of moderate or vigorous physical activity in last 12 months
Shoaff et al. [60]	USA	Prospective cohort	334	2	Prenatal	BMI z-score	PFOS PFOA PFHxS PFNA	Slope^a^	Maternal age, race, marital status, insurance status, income, education, parity, serum cotinine, depressive symptoms, mid-pregnancy BMI, food security, fruit/vegetable and fish consumption during pregnancy, prenatal vitamin use
Scinicariello et al. [59]	USA	Cross-sectional	600	3–11	Postnatal	BMI z-score	PFOS PFOA PFHxS PFNA	Slope^a^	Age, race/ethnicity, sex, poverty income ratio, serum cotinine, birthweight, maternal smoking during pregnancy
Timmermann et al. [52]	Denmark	Prospective cohort	499	8–10	Postnatal	BMI WC	PFOS PFOA	% Change	Sex, age, ethnicity, paternal income, fast food consumption, fitness, and WC was additionally adjusted for height
Vrijheid et al. [62]	Europe	Prospective cohort	1301	6.5–9	Postnatal	BMI z-score WC z-score	PFOS PFOA PFHxS PFNA PFUnDA	Slope Odds ratio	Sex, cohort, maternal education, maternal age, pre-pregnancy BMI, parity, parental country of birth, birth weight, breastfeeding duration
Yeung et al. [58]	USA	Prospective cohort	3111	3	Prenatal	BMI z-score	PFOS PFOA	Slope	Maternal age, pre-pregnancy BMI, education, race/ethnicity, private insurance status, infant sex

*PFOS* perfluorooctanesulfonic acid, *PFOA* perfluorooctanoic acid, *PFHxS* perflurohexanesulphonic acid, *PFNA* perfluorononanoic acid, *PFDA* perfluorodecanoic acid, *PFUnDA* perfluoroundecanoic acid, *BMI* body mass index, *WC* waist-circumference.

^a^Included in the meta-analysis.

Maternal serum and plasma median concentrations ranged from 4.52 to 33.80 ng/mL for PFOS, 1.00–5.60 ng/mL for PFOA, 0.20–2.40 ng/mL for PFHxS, 0.40–1.43 ng/mL for PFNA, and 0.26–0.37 ng/mL for PFDA. Child serum median concentrations ranged from 1.93 to 41.50 ng/mL for PFOS, 1.53–20.90 ng/mL for PFOA, 0.34–2.40 ng/mL for PFHxS, and 0.47–1.40 ng/mL for PFNA. Umbilical cord plasma and newborn bloodspot median concentrations ranged from 0.44 to 2.44 ng/mL for PFOS, 0.38–6.74 ng/mL for PFOA, 0.11–0.16 ng/mL for PFHxS, and 0.15–0.64 ng/mL for PFNA. Umbilical cord plasma and newborn bloodspot median concentrations ranged from 0.05 to 0.40 ng/mL for PFDA, PFUA, PFBS, PFDoA, and PFuNDA.

The study quality assessment using the OHAT criteria resulted in 13 studies ranked Tier 1, ten ranked Tier 2, and one ranked Tier 3. The details regarding the risk of bias assessment can be found in Table S1. A common strength was the exposure measurement using verified laboratory assessment methods, indicating that exposure assessments were valid and reliable. However, there was considerable variation in the outcome assessment and reporting method, including the type of measure of obesity and the age at which the measurement was conducted.

### Qualitative summary of 24 studies

A qualitative description of the included studies is provided in Table 2. Several studies reported little to no associations between exposure to various PFAS and childhood adiposity. For example, a 2013 study by Andersen et al. [50] used the Danish National Birth Cohort to assess prenatal exposure to PFOS and PFOA and BMI z-score, WC z-score, and the risk of being overweight at seven years old. They reported inverse associations of PFOS and PFOA with BMI and WC z-scores in girls and boys, but all the estimates were nonsignificant. There were no associations between PFOS and PFOA and the risk of overweight, and no dose-response relationships were observed. Likewise, a 2015 study by Hoyer et al. [31] using the INUENDO cohort in Greenland and Ukraine examined PFOS and PFOA and the risk of obesity but reported no significant associations.

**Table 2:** Qualitative summary of the included studies.

Time	Author	Measure	PFOS	PFOA	PFHxS	PFNA	PFBS	PFDA	PFuNDA
Prenatal	Andersen et al. [50]	BMIz	↔	↔	-	-	-	-	-
		WCz	↔	↔	-	-	-	-	-
	Bloom et al. [35]	BMIz	↔	↔	↔	↔	-	↔	↔
		WCz	↔	↔	↔	↔	-	↔	↑
	Braun et al. [29]	BMIz	↔	↑	↔	↔	-	-	-
		WC	↔	↑	↔	↔	-	-	-
	Chen et al. [55]	BMI	↔	↔	↔	↔	↔	↔	↔
		WC	↔	↔	↔	↔	↑	↔	↔
	Gross et al. [30]	Risk of overweight	↔	↔	↔	↔	-	-	-
	Hartman et al. [53]	BMI	↓	↓	↔	↔	-	-	-
		WC	↓	↓	↔	↔	-	-	-
	Horikoshi et al. [33]	BMIz-for-age	↑	↑	-	-	-	-	-
	Hoyer et al. [31]	Risk of obesity	↔	↔	-	-	-	-	-
	Lauritzen et al. [56]	BMIz	↑	↑	-	-	-	-	-
	Manzano-Salgado et al. [32]	BMIz - 4 years old	↔	↔	↔	↔	-	-	-
		WCz - 4 years old	↔	↔	↔	↔	-	-	-
		BMIz - 7 years old	↔	↔	↔	↔	-	-	-
		WCz - 7 years old	↔	↔	↔	↔	-	-	-
	Marks et al. [37]	BMIz-for-age	↔	↔	-	↔	-	-	-
	Martinsson et al. [51]	Risk of overweight	↔	↔	↔	↔	-	-	-
	Mora et al. [54]	BMI (girls only)	↔	↔	↔	↔	-	-	-
	Papadopoulou et al. [34]	WCz	↔	↔	↔	↔	-	-	-
	Schoaff et al. [60]	BMIz	↔	↔	↔	↔	-	-	-
	Yeung et al. [58]	BMIz	↓	↓	-	-	-	-	-
Postnatal	Canova et al. [36]	BMIz	↓	↔	↓				
	Fassier et al. [57]	BMIz	↓	↓	-	-	-	-	-
	Karlsen et al. [38]	BMIz	↑	↑	↔	↔	-	↔	-
	Lee et al. [61]	Height at two years	↓	↓	↓	↓	-	↓	-
	Papadopoulou et al. [34]	WCz	↔	↔	↔	↔	-	-	↔
	Pinney et al. [44]	BMIz Cincinnati	-	↔	-	-	-	-	-
		BMIz San Francisco	-	↔	-	-	-	-	-
	Scinicariello et al. [59]	BMIz	↔	↔	↔	↔	-	-	-
	Timmermann et al. [52]	BMI	↔	↔	-	-	-	-	-
		WC	↔	↔	-	-	-	-	-
	Vrijheid et al. [62]	BMIz	↓	↓	-	↓	-	-	↓

A case-control study in 2020 by Martinsson et al. [51] examined prenatal exposure to PFOS, PFOA, PFHxS, and PFNA, but found no significant associations with the risk of overweight at four years old. Another case-control study by Gross and colleagues [30] examined the same exposures as above in a cohort of Hispanic mother-infant pairs, however, they reported no statistically significant associations with the risk of being overweight at 18 months. A 2014 study by Timmermann et al. [52] examined PFOS, PFOA, BMI, and WC and observed no associations among overweight or normal weight children aged 8–10 years. However, they noted an interaction between PFOS and skinfold thickness, with skinfolds being thicker in girls than in boys, but these associations were also nonsignificant. Manzano-Salgado et al. [32] conducted a study using the IMNA Birth Cohort Study and examined PFOS, PFOA, PFHxS, and PFNA and cardiometabolic risk in children aged four and seven. They reported largely positive nonsignificant associations with BMI z-score and WC z-score for PFOS, PFOA, and PFNA for both time points; however, there were inverse associations with BMI and WC z-scores for those exposed to PFHxS, though nonsignificant. Similarly, a 2017 study by Hartman et al. [53] using the UK ALSPAC cohort examined prenatal exposure to PFOS, PFOA, PFHxS, and PFNA and body fatness in girls. They reported inverse associations of PFOS and PFOA exposure and BMI and WC, yet no significant associations with PFHxS and PFNA.

Furthermore, a prospective cohort study by Bloom et al. [35] examined several PFAS exposures, including PFOS, PFOA, PFHxS, PFNA, PFDA, and PFuNDA associated with childhood adiposity. Among mothers without obesity, all exposures, except PFuNDA, showed inverse associations with childhood BMI z-scores; nonetheless, there were no statistically significant findings. PFOS and PFHxS were inversely associated with WC z-scores, whereas PFOA, PFNA, and PFDA were positively associated. PFuNDA was the only exposure that was significantly positively associated with WC z-score. Associations differed for mothers with obesity, with all except PFOA and PFHxS showing inverse associations with BMI z-score, with PFOS and PFDA being significant. Associations with WC z-scores showed similar findings, with all exposures except PFOA and PFHxS having inverse associations, albeit nonsignificant. Another study by Mora et al. [54] reported positive, nonsignificant findings for prenatal exposure to PFOS, PFOA, PFHxS, and PFNA with BMI z-score in young girls. They also reported positive associations for all exposures except PFNA with WC changes; however, none were statistically significant.

Several studies found positive associations between PFAS exposures and childhood adiposity. For example, a prospective cohort study using the HOME cohort was conducted by Braun and colleagues [29] to assess exposure to PFOS, PFOA, PFHxS, and PFNA with changes in BMI z-score and WC. They reported positive associations between PFOA and BMI z-scores, WC, and body fat in eight-year-olds. Additionally, they observed non-linear associations wherein no additional increases in adiposity were detected among children born to women with PFOA concentrations surpassing the 50th percentile. All other associations were not statistically significant. Another study conducted in Shanghai, China by Chen et al. [55] found that prenatal exposure to PFBS was positively associated with WC in girls at age five; no other significant associations were observed. Likewise, Horikoshi et al. [33] and Lauritzen et al. [56] reported significant positive associations between PFOS and PFOA with BMI z-scores. Horikoshi et al. [33] observed larger effect sizes as the child ages until 66 months; at this point, the increase in BMI z-score was negligible. Lauritzen et al. [56] also observed increased odds of childhood overweight or obesity for each ln-unit increase in PFOS and PFOA concentrations. Similarly, Karlsen et al. [38] reported positive associations with PFOS and PFOA with BMI z-score and risk of overweight/obesity among Swedish and Norwegian children.

Conversely, several studies reported inverse associations between PFAS exposures and childhood adiposity measures. A 2021 cross-sectional study by Canova et al. [36] reported significant inverse associations of PFOS and PFHxS with BMI z-scores in girls and boys; there was an inverse association for PFOA and BMI z-score; however, this was nonsignificant. Likewise, another cross-sectional study conducted in 2019 by Fassler et al. [57] reported significant inverse associations between PFOS and PFOA and BMI z-scores in girls between the ages of six and eight. Similarly, Pinney et al. [44] used longitudinal data to examine the effects of serum concentrations of PFOA and BMI z-scores in girls from Cincinnati and San Francisco. The authors reported a significant inverse association between PFOA and BMI z-scores in girls from Cincinnati, but these effects were nonsignificant in the San Francisco cohort, and decreased with age. Also, a study using European cohorts by Vrijheid et al. [5] reported significant associations between early childhood exposures to PFOA, PFNA, and PFuNDA and BMI z-scores. Another prospective cohort study by Yeung et al. [58] observed a statistically significant inverse dose-response relationship between PFOA and BMI z-score among girls but not boys. Conversely, a 2020 study by Scinicariello et al. [59] using the NHANES cohort reported a statistically significant association between PFHxS and BMI z-score, but only in boys.

A prospective cohort study by Shoaff et al. [60] examined prenatal exposure to PFOA and its effect on infant growth rate. They observed inverse associations between PFOA and BMI z-score but did not observe any associations with growth rate in the first two years of life. A study by Papadopoulou et al. [34] examined prenatal and postnatal exposure to PFOS, PFOA, PFHxS, and PFNA and changes in WC as part of the HELIX project, a collaboration across six ongoing longitudinal population-based birth cohort studies in Europe. They reported significant inverse associations across all prenatal and postnatal exposures with changes in WC; however, prenatal exposure to PFNA was significantly positively associated with WC. Two studies by Lee et al. [61] and Marks et al. [37] examined PFAS exposures concerning changes in height at two years, height-for-age z-score, and BMI-for-age z-score. The prospective cohort study by Lee et al. [61] involved Korean children at two years of age and evaluated the effects of 15 PFAS exposures on growth parameters. They reported a statistically significant inverse association of PFHxS, PFOS, PFOA, PFNA, and PFDA with height at two years. Likewise, the study by Marks et al. [37] using the UK ALSPAC cohort examined prenatal exposure to eight PFAS chemicals and observed slight inverse associations with height-for-age and BMI-for-age z-scores; however, none were statistically significant.

### Results from the meta-analysis

There were 13 studies included in the meta-analysis (cohort *n* = 10, cross-sectional *n* = 3). Twelve studies examined PFOS exposure [29, 31–34, 36, 38, 50, 56, 57, 59, 60], 13 studies examined PFOA exposure [29, 31–34, 36, 38, 44, 50, 56, 57, 59, 60], eight studies examined PFHxS exposure [29, 32, 34, 36, 38, 57, 59, 60], and seven studies examined PFNA exposure [29, 32, 34, 38, 57, 59, 60]. There were too few studies to generate meaningful summary measures of association for PFDA, PFUnDA, PFDoA, PFUA, and PFBS exposures.

Two outcomes were examined in the meta-analysis, including changes in BMI z-scores and WC z-scores per 1-unit increase in log10-transformed PFAS exposure. Twelve studies examined BMI z-score changes, and four examined WC z-score changes. In addition, we stratified studies based on the timing of exposure to PFAS. Of the included studies in the meta-analysis, eight examined prenatal exposure to PFAS, and six examined postnatal (early childhood) exposures.

Eleven studies were excluded from the meta-analysis for various reasons. Several studies (*n* = 4) measured the association between PFAS and adiposity per IQR or SD increase in concentration. Still, these measures could not be used in the meta-analysis because the measures of association could not be converted to the log10 scale [35, 54, 58, 62]. Three studies used changes in BMI and WC but did not report any changes in BMI and WC z-scores, and thus, these studies were excluded as they could not be standardized to their z-score counterparts [52, 53, 55]. Some studies (*n* = 2) reported only odds ratios or relative risks for obesity; however, these measures used different reference points in defining obesity and could not be used in the meta-analysis [30, 51]. The remaining studies (*n* = 2) did not report usable associations for our meta-analysis (changes in height and weight and weight for age z-scores) [37, 61].

### PFOS exposure and adiposity

**Fig. 2:**
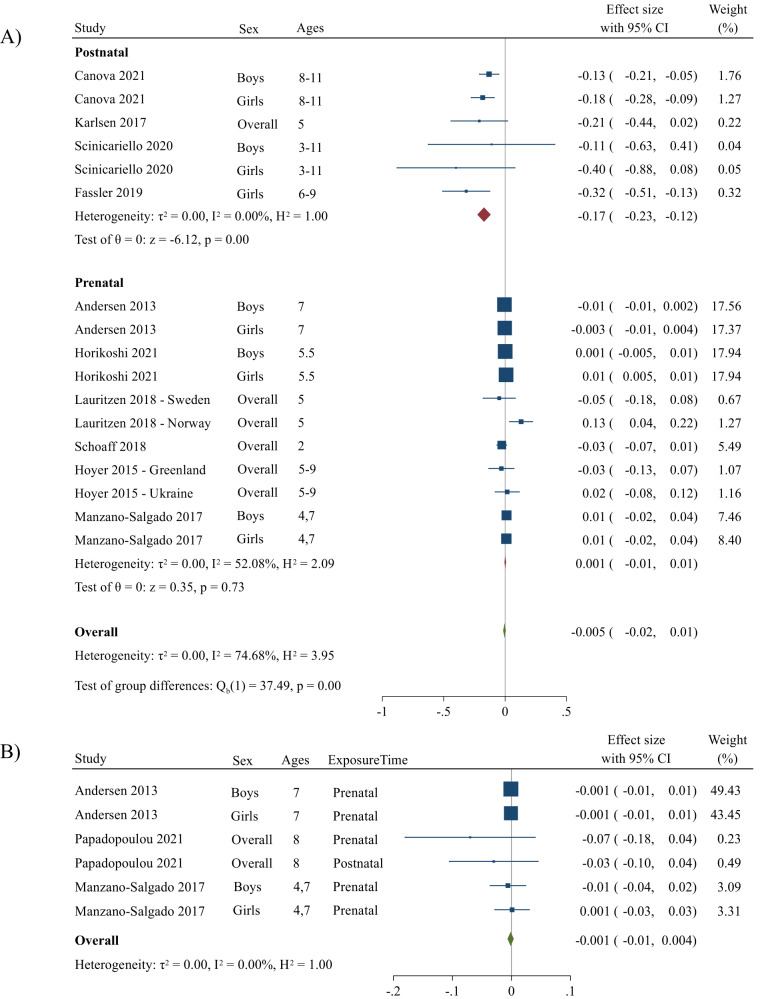
Forest plots including studies that report associations between PFOS and BMI z-score and WC z-score. Panel (**A**) represents the changes in BMI z-score per unit increase in log10-transformed PFOS concentration and panel (**B**) represents changes in WC z-score per unit increase in log10-transformed PFOS concentration. The weights represent the contribution of each study effect estimate to the overall meta-estimate.

The overall summary measure between PFOS exposure and change in BMI z-score per 1-unit increase in log10-transformed PFOS concentration was −0.005 (95% CI: −0.02, 0.01) (Fig. 2A). For prenatal exposure, the change in BMI z-score per 1-unit increase in log10-transformed PFOS concentration was 0.001 (95% CI: −0.01, 0.01), and for postnatal exposure, the change in BMI z-score per 1-unit increase in log10-transformed PFOS concentration was −0.17 (95% CI: −0.23, −0.12). A sensitivity analysis based on study quality was performed; when excluding Fassler et al. [57], the estimate per 1-unit increase in log10-transformed PFOS was −0.16 (−0.22, −0.1) for postnatal exposure, 0.001 (−0.01, 0.01) for prenatal exposure, and −0.003 (−0.01, 0.01) for overall exposure. No heterogeneity was detected when examining postnatal PFOS exposure and BMI z-score (*I*^*2*^ = 0.0%); however, when examining prenatal exposure, there was moderate heterogeneity (*I*^*2*^ = 52%). Overall, there was moderate to high heterogeneity (*I*^*2*^ = 75%); however, the absolute between-study heterogeneity was zero (τ^2^ = 0.0). The funnel plot showed moderate small-study effects such as publication bias (*p* = 0.0003), and when Fassler et al. was removed, the *p*-value remained significant (*p* = 0.005). When performing a Trim and Fill analysis, there was no change to the summary measure of effect (Fig. S1).

The summary measure between PFOS exposure and change in WC z-score per 1-unit increase in log10-transformed PFOS concentration was −0.001 (95% CI: −0.01, 0.004) (Fig. 2B). Only one study reported postnatal associations for change in WC z-scores; thus, we could not stratify by exposure time. When excluding the postnatal estimate (i.e., prenatal exposure only), the summary measure did not change. No relative or absolute between-study heterogeneity was seen when examining overall PFOS exposure and WC z-scores (*I*^*2*^ = 0.0%, τ^2^ = 0.0). There were no small-study effects for studies examining PFOS and WC z-scores (*p* = 0.2) (Fig. S2).

### PFOA exposure and adiposity

**Fig. 3:**
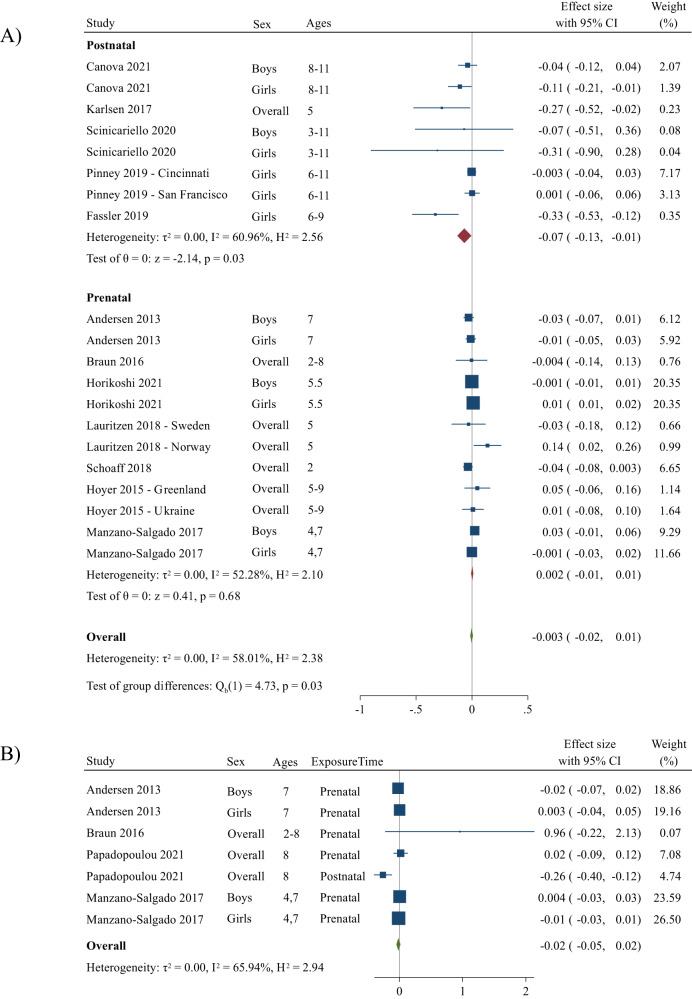
Forest plots including studies that report associations between PFOA and BMI z-score and WC z-score. Panel (**A**) represents changes in BMI z-score per unit increase in log10-transformed PFOA concentration and panel (**B**) represents changes in WC z-score per unit increase in log10-transformed PFOA concentration. The weights represent the contribution of each study effect estimate to the overall meta-estimate.

The overall summary measure between PFOA exposure and change in BMI z-score per 1-unit increase in log10-transformed PFOA concentration was −0.003 (95% CI: −0.02, 0.01) (Fig. 3A). For prenatal exposure, the change in BMI z-score per 1-unit increase in log10-transformed PFOA concentration was 0.002 (95% CI: −0.01, 0.01), and for postnatal exposure, the change in BMI z-score per 1-unit increase in log10-transformed PFOA concentration was −0.07 (95% CI: −0.13, −0.01) and statistically significant. When excluding Fassler et al. [57], the postnatal estimate per 1-unit increase in log10-transformed PFOA was −0.04 (−0.1, 0.01) and was no longer statistically significant. The estimate for prenatal exposure was 0.002 (−0.01, 0.01), and the overall summary estimate was −0.001 (−0.01, 0.01) per 1-unit increase in log10-transformed PFOA exposure. Moderate heterogeneity was seen when examining postnatal PFOA exposure and BMI z-scores (*I*^*2*^ = 61%) and prenatal exposure (*I*^*2*^ = 52%). Overall, there was moderate heterogeneity across studies (*I*^*2*^ = 58%); however, the absolute between-study heterogeneity was zero (τ^2^ = 0.0). The funnel plot showed moderate small-study effects such as publication bias (*p* = 0.02), but when Fassler et al. [57] was excluded, this was attenuated (*p* = 0.1). There was no change to the summary effect size when performing a Trim and Fill analysis (Fig. S3).

The summary measure between PFOA exposure and change in WC z-score per 1-unit increase in log10-transformed PFOA concentration was −0.02 (95% CI: −0.05, 0.02) (Fig. 3B). Only one study reported postnatal associations for change in WC z-scores; thus, we could not stratify by exposure time. When excluding the postnatal estimate (i.e., prenatal exposure only), the summary measure was −0.01 (95% CI: −0.02, 0.01). There was moderate heterogeneity when examining overall PFOA exposure and WC z-scores (*I* ^*2*^ = 66%), but the absolute between-study heterogeneity was zero (τ^2^ = 0.0). There were no small-study effects for studies examining PFOA and WC z-scores (*p* = 0.8) (Fig. S4).

### PFHxS exposure and adiposity

**Fig. 4:**
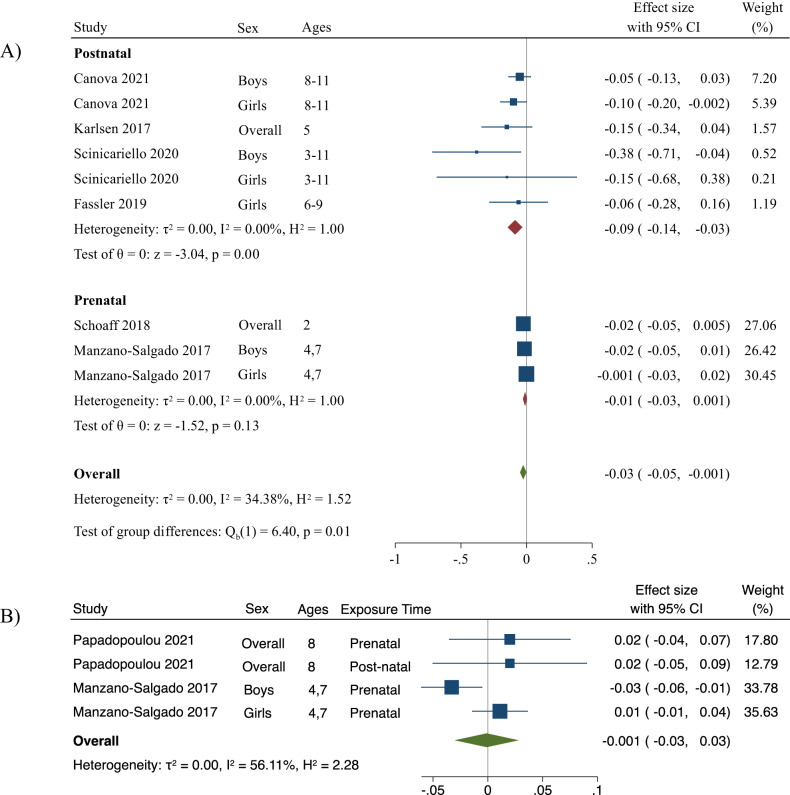
Forest plots including studies that report associations between PFHxS and BMI z-score and WC z-score. Panel (**A**) represents changes in BMI z-score per log10 increase in PFHxS concentration and panel (**B**) represents changes in WC z-score per unit increase in log10-transformed PFHxS concentration. The weights represent the contribution of each study effect estimate to the overall meta-estimate.

The overall summary measure between PFHxS exposure and change in BMI z-score per 1-unit increase in log10-transformed PFHxS concentration was −0.03 (95% CI: −0.05, −0.001) (Fig. 4A). For prenatal exposure, the change in BMI z-score per 1-unit increase in log10-transformed PFHxS concentration was −0.01 (95% CI: −0.03, 0.001), and for postnatal exposure, the change in BMI z-score per 1-unit increase in log10-transformed PFHxS concentration was -0.09 (95% CI: −0.14, −0.03) and statistically significant. There were no changes in the estimates when Fassler et al. [57] was excluded. No relative between-study heterogeneity was seen when examining postnatal or prenatal PFHxS exposure and BMI z-scores (*I*^*2*^ = 0%). Overall, there was low heterogeneity across studies (*I*^*2*^ = 34%), and the absolute between-study heterogeneity was zero (τ^2^ = 0.0). The funnel plot showed moderate small study effects (*p* = 0.004) and remained when Fassler et al. [57] was excluded (*p* = 0.003). When performing a Trim and Fill analysis, there was no change to the effect size (Fig. S5).

The summary measure between PFHxS exposure and change in WC z-score per 1-unit increase in log10-transformed PFHxS concentration was −0.001 (95% CI: −0.03, 0.03) (Fig. 4B). Only one study reported postnatal associations for change in WC z-scores; thus, we did not stratify by exposure time. When excluding the postnatal estimate (i.e., prenatal exposure only), the summary measure was −0.004 (95% CI: −0.04, 0.03). There was moderate heterogeneity when examining overall PFHxS exposure and WC z-scores (*I*^*2*^ = 56%) and no absolute between-study heterogeneity (τ^2^ = 0.0). There were no small-study effects for studies examining PFHxS and WC z-scores (*p* = 0.4), but when Fassler et al. [57] was excluded, there were small-study effects present (*p* = 0.003) (Fig. S6).

### PFNA exposure and adiposity

**Fig. 5:**
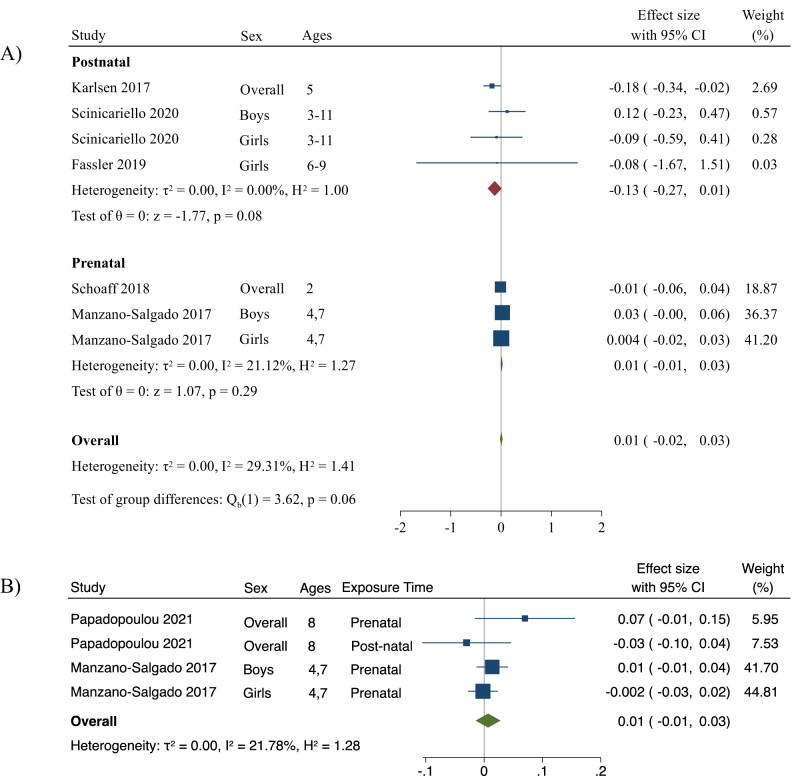
Forest plots including studies that report associations between PFNA and BMI z-score and WC z-score. Panel (**A**) represents changes in BMI z-score per unit increase in log10-transformed PFNA concentration and panel (**B**) represents changes in WC z-score per unit increase in log10-transformed PFNA concentration. The weights represent the contribution of each study effect estimate to the overall meta-estimate.

The overall summary measure between PFNA exposure and change in BMI z-score per 1-unit increase in log10-transformed PFNA concentration was 0.01 (95% CI: −0.02, 0.03) (Fig. 5A). For prenatal exposure, the change in BMI z-score per 1-unit increase in log10-transformed PFNA concentration was 0.01 (95% CI: −0.01, 0.03), and for postnatal exposure, the change in BMI z-score per 1-unit increase in log10-transformed PFNA concentration was −0.13 (95% CI: −0.27, 0.01). When excluding Fassler et al., the postnatal estimate was −0.09 (−0.2, −0.03) and statistically significant. The prenatal estimate per 1-unit increase in log10-transformed PFNA was −0.01 (−0.03, 0.004), and the overall estimate was −0.03 (−0.05, −0.001) and statistically significant. No relative heterogeneity was seen when examining postnatal PFNA exposure and BMI z-scores (*I*^*2*^ = 0%), and low heterogeneity for prenatal exposure (*I*^*2*^ = 21%). Overall, there was low heterogeneity across studies (*I*^*2*^ = 29%) and no absolute between-study heterogeneity (τ^2^ = 0.0). In addition, there was no publication bias for studies examining PFNA and BMI z-scores (*p* = 0.3) (Fig. S7), and there were no changes when Fassler et al. [57] was excluded.

The summary measure between PFNA exposure and change in WC z-score per 1-unit increase in log10-transformed PFNA concentration was 0.01 (95% CI: −0.01, 0.03) (Fig. 5B). Only one study reported postnatal associations for change in WC z-scores; thus, we did not stratify by exposure time. When excluding the postnatal estimate (i.e., prenatal exposure only), the summary measure did not change. Low relative heterogeneity (*I*^*2*^ = 22%) and no absolute between-study heterogeneity (τ^2^ = 0.0) was seen across studies examining overall PFNA exposure and WC z-scores. No small-study effects were observed (*p* = 0.7) (Fig. S8).

## Discussion

Overall, there were 24 studies included in our systematic review examining pediatric obesity following PFAS exposures, and summary measures were derived using data from 13 epidemiological studies. Overall exposures to PFOS and PFOA per 1-unit increase in log10-transformed concentration resulted in non-statistically significant inverse associations with BMI z-scores; however, exposure to PFHxS was inversely, significantly associated with BMI z-scores. Conversely, overall exposure to PFNA was non-significantly positively associated with changes in BMI z-scores but subsequently became inversely significantly associated following the exclusion of Fassler et al. [57]. There were no statistically significant associations between PFAS exposures and WC z-scores.

Our summary measures were similar to those reported by Stratakis et al. [28], as we both report positive but non-statistically significant findings for prenatal exposure to PFOS and PFOA and changes in BMI z-scores. Our inverse, non-statistically significant summary measure for WC and prenatal PFOS exposure is also consistent with Stratakis et al. [28]; however, there are differences for PFOA exposure where we report a non-significant inverse relationship, contrasting their non-significant positive estimate. In addition to examining PFOS and PFOA exposures, we also considered prenatal exposure to PFHxS and PFNA, which yielded non-significant inverse and positive associations with BMI z-scores, respectively.

There were differences in the association’s direction when stratifying by exposure time. Prenatal exposure (except for PFHxS) increased BMI z-scores, though nonsignificant. Conversely, postnatal exposures to all examined PFAS were inversely related to BMI z-scores. Associations for postnatal PFOS and PFHxS exposure and BMI z-scores were statistically significant; PFOA exposure became nonsignificant following the exclusion of Fassler et al. [57], whereas PFNA exposure became significantly associated with BMI z-score after exclusion. There were no statistically significant associations between prenatal PFAS exposure and WC z-score. These results indicate differences in the effect of PFAS exposure according to the timing of exposure. However, these results should be interpreted cautiously due to the small number of studies. These differences may be due to several factors, including the timing of measurement of exposure and outcome, the method of measurement, and the overall concentration levels of PFAS. There are apparent differences in the need to target the mother during pregnancy and the child in early life to reduce these exposures when considering health prevention methods.

The biological mechanism through which PFAS exposure affects childhood growth and obesity remains unclear. A proposed mechanism of PFAS toxicity is the PFAS-mediated activation of the PPARα [15]. PPARs are involved in modulating lipid and glucose metabolism and the differentiation of adipocytes (lipid storage cells) during development [63]. Furthermore, PFAS-activated PPAR signaling may contribute to increased adiposity and risk of obesity in children via increased inflammation and adipogenesis [64]. Animal studies have shown that the effects of PFAS exposure act through different mechanisms depending on the type and concentration of exposure [65, 66]. This suggests that there may be differences in the health effects under different circumstances, but it is unclear how these effects vary across populations. Furthermore, PFAS have been identified as thyroid-disrupting chemicals, resulting in the dysregulation of the hypothalamic-pituitary-thyroid axis, synthesis of hormones, and metabolism [67]. Thyroid hormones play a critical part in normal growth and development; thus, thyroid function changes may increase the risks of abnormal growth and adiposity during childhood [68].

Among the included studies, the covariates most adjusted for included maternal pre-pregnancy BMI, maternal age, parental race/ethnicity, socioeconomic measures (most common was the level of education and household income), maternal smoking status before and during pregnancy, and parity. Other covariates included gestational age, birth weight, and duration of breastfeeding, all of which have been related to childhood growth and development [50]. All studies accounted for maternal age except for Martinsson et al. [51]. According to a cross-sectional study conducted in Finland, there was a higher risk of obesity at the age of seven among children born to mothers over 40 [69]. Consequently, failing to consider maternal age in the analysis may create a misleading perception of a stronger connection between PFAS and childhood obesity. Eight studies used directed acyclic graphs to identify relevant confounders [31, 34, 36, 38, 56, 60, 62, 69].

Interestingly, the study conducted by Manzano-Salgado et al. [32] was the only study to measure and adjust for maternal serum albumin, which is a binding site for PFAS in the blood [70], as well as maternal glomerular filtration rate, which may confound the association between PFAS and fetal growth [71]. A 2020 study [72] reported that albumin, found in blood plasma and serum, is the major carrier protein for several PFAS chemicals. Failure to adjust for these measures may result in overestimating the effect of PFAS and childhood anthropometry. Five studies considered the child’s physical activity or fitness levels as potential confounding factors [32, 36, 52, 53]. Recent work has examined the relationship between PFAS and physical activity and fitness levels [73, 74], as these may modify the associations between PFAS and anthropometric outcomes. Still, more research is needed to draw accurate conclusions. One study reported that increasing PFOA concentrations were associated with worse cardiometabolic risk scores for children with low activity but not children with high activity levels [73]. Another study reported no statistically significant associations between PFAS and physical activity; however, they reported consistently significant associations between PFDA and fitness levels [74]. Dietary intake was considered as a covariate in several studies [29, 32, 36, 38, 51–53, 60] however, these measures were very heterogenous according to the type of food examined (i.e., fruits, vegetables, fish, fast food), type of assessments used (i.e., dietary intake scales, 24-hour intake recalls), and whether the parent or child completed the dietary questionnaire. Dietary intake is one of the major contributors to PFAS exposure [75], and several studies have quantified PFAS concentrations in foods such as fish, dairy, and fast food [76, 77]. Since dietary intake is associated with PFAS concentration and anthropometric measures, it presents as a major confounder and should be conditioned on in future analyses.

Our quality assessment found that 19/24 studies had good or adequate information regarding confounding variables. In contrast, there was deficient information available in four studies, with one study (Fassler et al., 2019) having critically deficient information available.

There are some strengths associated with this study. First, we conducted a comprehensive search of available literature across multiple databases, which allowed for a high probability of identification of all potentially eligible studies published up to 2022. We recognize that over a year has passed since conducting the literature search; however, to our knowledge, additional eligible studies have not been published since our search was conducted. Next, the quality assessment of all included papers found that many studies were high (Tier 1, 13/24 studies) or moderate (Tier 2, 10/24 studies), increasing the summary estimations’ quality. Another strength is that only low to moderate heterogeneity was seen in most subgroups, although the small number of studies makes it challenging to examine and draw accurate conclusions.

Conversely, one limitation of our study was the inability to run subgroup analyses examining sex differences, as there were insufficient studies. Often, there are differences between males and females when examining adiposity and the risk of obesity [78], which may limit our results’ validity. Additionally, we could not stratify the results based on child ages, which may also affect the summary estimates, as there have been differences in early childhood versus middle childhood growth development [32]. Furthermore, there are large variations in food intake throughout childhood, as young children depend on their caregivers for nutrition, but older children can make more independent choices regarding consumption. In previous work using the HOME study, prenatal serum PFOA concentrations were associated with rapid adiposity gains from 2 to 8 years of age and excess adiposity at eight years; however, there was no association with growth in the first two years of life [29, 60]. These findings suggest that PFAS exposure may have differential impacts on adiposity throughout different stages of growth and development. Future research should examine childhood growth trajectories with PFAS exposures to better understand PFAS-related adiposity changes throughout childhood and adolescence.

The studies included in our review varied substantially for exposure and outcome assessments, making it difficult to perform a meta-analysis. Factors that complicated undertaking meta-analyses were differences in the age of participants when samples were collected, different sampling media (e.g., urine versus maternal/childhood serum), routes of exposure (i.e., maternal, diet, ambient air, contaminated water), and outcome measurements (i.e., BMI and WC z-scores, weight-for-length and age z-scores, changes in weight and height). Using accurate and reliable methods to assess body composition is essential to investigate excess adiposity and its associated health consequences. Several anthropometric measures are used when assessing pediatric obesity, including BMI, WC, changes in weight and height, and skinfold thickness, among others [79]. BMI is highly correlated with total body fat at high levels, and thus, specific cut-points are used to define excess adiposity and obesity in childhood [80]. BMI z-scores serve as standardized measures, enabling comparisons among children of varying ages and genders. Observational studies have established clinically significant changes in BMI z-scores, indicating that a reduction of 0.15–0.25 is associated with decreases in cardiometabolic risk factors [81, 82]. Moreover, an expert panel in Germany has determined that a decrease of 0.20 in BMI z-score is akin to a 5% weight reduction [83]. Therefore, a decrease in BMI z-score ranging from 0.20 to 0.25 holds clinical significance [82].

All the above factors underscore the importance of reaching a consensus on the analytical methods of assessing childhood obesity concerning environmental exposures. A more standardized approach is needed to measure and model the associations between PFAS exposure and pediatric obesity.

Publication bias is an important consideration when conducting meta-analysis. This bias arises because studies with positive findings are more likely to be published than those with null results. We found some evidence of publication bias for studies evaluating PFOS, PFOA, PFHxS, and changes in BMI z-scores. However, there were no changes in the effect estimates after adjusting for this bias with Trim and Fill analyses. In addition, we found no evidence of publication bias for the other outcomes considered; however, we recognize we have limited power to assess this bias given the small number of studies.

## Conclusion

Overall, we found inverse non-statistically significant associations between PFOS, PFOA, and PFHxS exposure and changes in BMI and WC z-scores. Exposures to PFNA showed positive, non-statistically significant associations for changes in both obesity measures. The timing of exposure seems to explain heterogeneity in summary measures, as postnatal exposure appears to have more of a protective effect with statistically significant associations. In contrast, prenatal exposure appears to increase the risks of pediatric obesity with positive but non-statistically significant associations. Current evidence suggests that prenatal PFAS exposure impairs proper fetal growth and may increase the risk for pediatric obesity; however, our summary results suggest that the effects depend on the time of exposure and vulnerability. Any conclusions should be interpreted cautiously due to the small number of studies. Future research is warranted to explore further the associations between pediatric obesity and various PFAS exposures, including multi-exposure models, temporal differences in exposure, outcome assessments, potential dose-response relationships, and a deeper understanding of the biological mechanisms related to prenatal and postnatal exposures.

### Supplementary information

Supplementary material
