# Supplementary material for: Exposure to perfluoroalkyl and polyfluoroalkyl substances and pediatric obesity: a systematic review and meta-analysis
Source: Int J Obes (Lond). 2023 Oct 31;48(2):131–46. doi: 10.1038/s41366-023-01401-6 (PMC10824662; doi:10.1038/s41366-023-01401-6)
Supplement: Supplementary file 1 — Supplementary material [file 41366_2023_1401_MOESM1_ESM.docx]

**Supplementary material**

This file contains funnel plots assessing small-study effects such as publication bias for each of the PFAS chemicals explored, and for both BMI and WC z-scores. There are also the details regarding the study quality assessment using the OHAT criteria. Literature search strategy is provided at the end.

Figure S1. Funnel plot of risk estimates from studies that examined the association between PFOS exposure and changes in BMI z-score. p = 0.0053

Figure S2. Funnel plot of risk estimates from studies that examined the association between PFOS exposure and changes in WC z-score. p = 0.32

Figure S3. Funnel plot of risk estimates from studies that examined the association between PFOA exposure and changes in BMI z-score. p = 0.038

Figure S4. Funnel plot of risk estimates from studies that examined the association between PFOA exposure and changes in WC z-score. p = 0.84

Figure S5. Funnel plot of risk estimates from studies that examined the association between PFHxS exposure and changes in BMI z-score. p = 0.0045

Figure S6. Funnel plot of risk estimates from studies that examined the association between PFHxS exposure and changes in WC z-score. p = 0.46

Figure S7. Funnel plot of risk estimates from studies that examined the association between PFNA exposure and changes in BMI z-score. p = 0.32

Figure S8. Funnel plot of risk estimates from studies that examined the association between PFNA exposure and changes in WC z-score. p = 0.56

Figure S1. Funnel plot of risk estimates from studies that examined the association between PFOS exposure and changes in BMI z-score. P=0.0003.

Figure S2. Funnel plot of risk estimates from studies that examined the association between PFOS exposure and changes in WC z-score. P=0.2.

Figure S3. Funnel plot of risk estimates from studies that examined the association between PFOA exposure and changes in BMI z-score. P=0.02.

Figure S4. Funnel plot of risk estimates from studies that examined the association between PFOA exposure and changes in WC z-score. P=0.8.

Figure S5. Funnel plot of risk estimates from studies that examined the association between PFHxS exposure and changes in BMI z-score. P=0.004.

Figure S6. Funnel plot of risk estimates from studies that examined the association between PFHxS exposure and changes in WC z-score. P=0.4.

Figure S7. Funnel plot of risk estimates from studies that examined the association between PFNA exposure and changes in BMI z-score. P=0.3.

Figure S8. Funnel plot of risk estimates from studies that examined the association between PFNA exposure and changes in WC z-score. P=0.7.

Table S1. Study quality assessment using OHAT criteria.

| **Author** | **Year** | **Participant Selection** | **Confounding** | **Attrition/Exclusion** | **Exposure Measurement** | **Outcome Assessment** | **Outcome Reporting** | **Statistical Methods** | **Overall Study Confidence** |
| --- | --- | --- | --- | --- | --- | --- | --- | --- | --- |
| Andersen et al. | 2013 | ++ | ++ | + | ++ | + | ++ | + | Tier 2 |
| Bloom et al. | 2022 | + | + | + | ++ | ++ | ++ | ++ | Tier 2 |
| Braun et al. | 2016 | ++ | ++ | + | ++ | ++ | ++ | ++ | Tier 1 |
| Canova et al. | 2021 | ++ | ++ | + | ++ | + | ++ | ++ | Tier 1 |
| Chen et al. | 2019 | + | + | + | ++ | ++ | ++ | + | Tier 2 |
| Fassler et al. | 2019 | + | -- | + | ++ | ++ | + | + | Tier 3 |
| Gross et al. | 2020 | - | + | + | ++ | + | - | + | Tier 2 |
| Hartman et al. | 2017 | + | - | - | ++ | ++ | + | + | Tier 2 |
| Horikoshi et al. | 2021 | ++ | + | ++ | ++ | ++ | ++ | ++ | Tier 1 |
| Hoyer et al. | 2015 | ++ | ++ | + | ++ | + | + | ++ | Tier 1 |
| Karlsen et al. | 2017 | + | ++ | - | ++ | ++ | ++ | ++ | Tier 1 |
| Lauritzen et al. | 2018 | + | ++ | + | ++ | ++ | ++ | ++ | Tier 1 |
| Lee et al. | 2018 | ++ | - | ++ | ++ | ++ | + | + | Tier 2 |
| Manzano-Salgado et al. | 2017 | ++ | ++ | + | ++ | ++ | ++ | ++ | Tier 1 |
| Marks et al. | 2021 | ++ | - | - | ++ | ++ | + | ++ | Tier 2 |
| Martinsson et al. | 2020 | ++ | - | + | ++ | - | + | ++ | Tier 2 |
| Mora et al. | 2017 | ++ | ++ | + | ++ | ++ | ++ | ++ | Tier 1 |
| Papadopoulou et al. | 2021 | ++ | ++ | + | ++ | ++ | + | ++ | Tier 1 |
| Pinney et al. | 2019 | ++ | + | + | ++ | ++ | ++ | ++ | Tier 1 |
| Schoaff et al. | 2018 | ++ | ++ | - | ++ | ++ | ++ | ++ | Tier 1 |
| Scinicariello et al. | 2020 | ++ | + | - | + | + | ++ | ++ | Tier 1 |
| Timmermann et al. | 2014 | + | + | + | + | ++ | ++ | ++ | Tier 2 |
| Vrijheid et al. | 2020 | ++ | ++ | ++ | ++ | ++ | ++ | ++ | Tier 1 |
| Yeung et al. | 2019 | + | + | + | ++ | + | ++ | ++ | Tier 2 |

(++) represents a rating of “good” for the cell; (+) represents a rating of “adequate” for the cell; (-) represents a rating of “deficient” for the cell; (--) represents a rating of “critically deficient” for the cell

Supplementary Table 1. Search strategy used to identify studies

| MEDLINE, Embase, PsychInfo, Cochrane Central (1639 HITS) |
| --- |
| 1. (PFAS or PFBS or PFHxS or PFOS or PFHxA or PFOA or PFNA or PFDA or Perfluorinated or (perfluorooctane adj1 sulfonate) or perfluorooctanoate or (polyfluoroalkyl adj1 compounds) or (Polyfluoroalkyl adj1 chemicals) or (Perfluorinated adj1 chemicals) or (Perfluorooctanoic adj1 acid) or (perfluorooctane adj1 sulfonic adj1 acid) or (perfluorinated adj1 acid) or fluorocarbons or (Perfluorinated adj1 alkyl adj1 substances) or (Perfluorohexane adj1 sulfonate) or (perfluoroalkyl adj1 acids) or (fluorinated adj1 organic adj1 compounds) or (endocrine adj1 disrupting adj1 chemicals) or (metabolism adj1 disrupting adj1 chemicals) or (endocrine adj1 disruptors) or obesogen* or (environmental adj1 chemicals)).ti,ab. |
| 1. (chemical adj1 exposure).ti,ab. |
| 1. endocrine disruptor/ |
| 1. environmental exposure/ |
| 1. 1 or 2 or 3 or 4 |
| 1. (Adiposity or (body adj1 mass adj1 index) or (waist adj1 circumference) or (waist adj1 to adj1 hip adj1 ratio) or obese or obesity or overweight or obesogen*).ti,ab. |
| 1. obesity/ |
| 1. body mass/ |
| 1. waist circumference/ |
| 1. waist hip ratio/ |
| 1. 6 or 7 or 8 or 9 or 10 |
| 1. (Childhood or prenatal).ti,ab. |
| 1. child/ |
| 1. infant/ |
| 1. 12 or 13 or 14 |
| 1. 5 and 11 and 15 |
| 1. animal/ |
| 1. (animals or animal).ti. |
| 1. 17 and 18 |
| 1. 16 not 19 |
| 1. adults/ or adolescent/ or (adult* or adolescen* or teen*).ti,ab. |
| 1. 20 not 21 |
| 1. clinical trial/ or "clinical trial (topic)"/ or clinical trial protocol/ or clinical trial registry/ or (Ecological adj1 (study or research or methodology or design)).ti,ab. |
| 1. 22 not 23 |
| 1. limit 24 to human |
| 1. limit 25 to yr="2000 -Current" |
|  |
|  |
| Web of Science (876 HITS) |
| 1. (TI=(PFAS OR PFBS OR PFHxS OR PFOS OR PFHxA OR PFOA OR PFNA OR PFDA OR Perfluorinated OR (perfluorooctane NEAR/1 sulfonate ) OR perfluorooctanoate OR (polyfluoroalkyl NEAR/1 compounds ) OR (Polyfluoroalkyl NEAR/1 chemicals ) OR (Perfluorinated NEAR/1 chemicals ) OR (Perfluorooctanoic NEAR/1 acid ) OR (perfluorooctane NEAR/1 sulfonic NEAR/1 acid ) OR (perfluorinated NEAR/1 acid ) OR fluorocarbons OR (Perfluorinated NEAR/1 alkyl NEAR/1 substances ) OR (Perfluorohexane NEAR/1 sulfonate ) OR (perfluoroalkyl NEAR/1 acids ) OR (fluorinated NEAR/1 organic NEAR/1 compounds ) OR (endocrine NEAR/1 disrupting NEAR/1 chemicals ) OR (metabolism NEAR/1 disrupting NEAR/1 chemicals ) OR (endocrine NEAR/1 disruptors ) OR obesogen* OR (environmental NEAR/1 chemicals )) OR AB=(PFAS OR PFBS OR PFHxS OR PFOS OR PFHxA OR PFOA OR PFNA OR PFDA OR Perfluorinated OR (perfluorooctane NEAR/1 sulfonate ) OR perfluorooctanoate OR (polyfluoroalkyl NEAR/1 compounds ) OR (Polyfluoroalkyl NEAR/1 chemicals ) OR (Perfluorinated NEAR/1 chemicals ) OR (Perfluorooctanoic NEAR/1 acid ) OR (perfluorooctane NEAR/1 sulfonic NEAR/1 acid ) OR (perfluorinated NEAR/1 acid ) OR fluorocarbons OR (Perfluorinated NEAR/1 alkyl NEAR/1 substances ) OR (Perfluorohexane NEAR/1 sulfonate ) OR (perfluoroalkyl NEAR/1 acids ) OR (fluorinated NEAR/1 organic NEAR/1 compounds ) OR (endocrine NEAR/1 disrupting NEAR/1 chemicals ) OR (metabolism NEAR/1 disrupting NEAR/1 chemicals ) OR (endocrine NEAR/1 disruptors ) OR obesogen* OR (environmental NEAR/1 chemicals ))) |
| 1. (TI=(chemical NEAR/1 exposure ) OR AB=(chemical NEAR/1 exposure )) |
| 1. ALL="endocrine disruptor" |
| 1. ALL="environmental exposure" |
| 1. #1 OR #2 OR #3 OR #4 |
| 1. (TI=(Adiposity OR (body NEAR/1 mass NEAR/1 index ) OR (waist NEAR/1 circumference ) OR (waist NEAR/1 to NEAR/1 hip NEAR/1 ratio ) OR obese OR obesity OR overweight OR obesogen* ) OR AB=(Adiposity OR (body NEAR/1 mass NEAR/1 index ) OR (waist NEAR/1 circumference ) OR (waist NEAR/1 to NEAR/1 hip NEAR/1 ratio ) OR obese OR obesity OR overweight OR obesogen* )) |
| 1. ALL=obesity |
| 1. ALL="body mass" |
| 1. ALL="waist circumference" |
| 1. ALL="waist hip ratio" |
| 1. #6 OR #7 OR #8 OR #9 OR #10 |
| 1. (TI=(Childhood OR prenatal ) OR AB=(Childhood OR prenatal )) |
| 1. ALL=child |
| 1. ALL=infant |
| 1. #12 OR #13 OR #14 |
| 1. #5 AND #11 AND #15 |
| 1. ALL=animal |
| 1. TI=(animals OR animal ) |
| 1. #17 AND #18 |
| 1. #16 NOT #19 |
| 1. ALL=adults OR ALL=adolescent OR (TI=(adult* OR adolescen* OR teen* ) OR AB=(adult* OR adolescen* OR teen* )) |
| 1. #20 NOT #21 |
| 1. ALL="clinical trial" OR ALL="clinical trial (topic)" OR ALL="clinical trial protocol" OR ALL="clinical trial registry" OR (TI=(Ecological NEAR/1 (study OR research OR methodology OR design )) OR AB=(Ecological NEAR/1 (study OR research OR methodology OR design ))) |
| 1. #22 NOT #23 |
| 1. 2000-01-01 to 2022-01-24 |
|  |

Total pre-duplicate removal = 2 515

Total post-duplicate removal = 1 754
